# Exploring the Influencing Factors of Health Literacy among Older Adults: A Cross-Sectional Survey

**DOI:** 10.3390/medicina56070330

**Published:** 2020-07-02

**Authors:** Hsiao-Ting Chiu, Han-Wei Tsai, Ken N. Kuo, Angela Y.M. Leung, Yao-Mao Chang, Pi-Hsia Lee, Wen-Hsuan Hou

**Affiliations:** 1 Master’s Program in Long-Term Care, College of Nursing, Taipei Medical University, Taipei 110, Taiwan; chiu8782@yahoo.com.tw (H.-T.C.); hangwei.t@gmail.com (H.-W.T.); 2 Department of Rehabilitation, Xinwu Branch, Taoyuan General Hospital, Ministry of Health and Welfare, Taoyuan 327, Taiwan; 3 Cochrane Taiwan, Taipei Medical University, Taipei 110, Taiwan; kennank@aol.com; 4 Centre for Gerontological Nursing, School of Nursing, The Hong Kong Polytechnic University, Hong Kong, China; angela.ym.leung@polyu.edu.hk; 5 Health Policy and Care Research Center, College of Public Health, Taipei Medical University, Taipei 110, Taiwan; yaomaoc@gmail.com; 6 School of Nursing, College of Nursing, Taipei Medical University, Taipei 110, Taiwan; pihsia@tmu.edu.tw; 7 Department of Physical Medicine and Rehabilitation, Taipei Medical University Hospital, Taipei 110, Taiwan; 8 Graduate Institute of Clinical Medicine, College of Medicine, Taipei Medical University, Taipei 110, Taiwan

**Keywords:** health literacy, older adults, dominant spoken dialect, influencing factors of health literacy

## Abstract

*Background and Objectives:* To investigate the health literacy (HL) among older adults in Taiwan, we referenced an existing integrated model of HL to confirm the influencing factors of HL in older adults. We propose this study to examine the personal, situational, and socioenvironmental factors influencing HL among older adults. *Materials and Methods*: A cross-sectional survey was conducted at a district hospital and affiliated community center in northern Taiwan from August 2016 to May 2017. This study used the Mandarin Chinese version of the European Health Literacy Survey Questionnaire (EU-Q47). We designed three models based on the three domains of HL. Model 1 assesses personal factors. Model 2 incorporates situational factors. Model 3 adds the socioenvironmental factor. *Results:* We recruited 161 participants aged over 65 years. Most adults in this study had limited overall HL. The final regression model revealed that age >85 years, unknown insurance status, and dominant spoken dialect of Hakka or Taiwanese were significantly associated with higher scores of HL. *Conclusions*: Our study results may help clinicians with early identification of older adults at high risk for poor HL and help health administrators establish geriatric policies and health education plans.

## 1. Introduction

Health literacy (HL) has been defined as the set of skills required to obtain, process, and understand basic health information in medical situations [1]. It extends to the ability to apply information when making decisions regarding one’s health and life quality [2]. Inadequate HL is associated with impaired healthcare choices leading to poor quality-of-care [3]. HL also affects perception of illness, which guides self-management and medical treatment [4]. Most assessments of HL focus on access to, understanding of, and use of health information in medical settings and overlook cognitive skills, communication skills, and the ability to analyze the value of messages that may assist in personal health management [5]. The European Health Literacy Consortium proposed a conceptual model of HL encompassing three components: the core concept of HL, antecedents, and consequences of HL. The antecedents of HL include three types of determinant (personal, situational, and socioenvironmental). Personal factors and situational factors are proximal factors; the former include gender, age, education, occupation, income, and insurance, while the later include marital status, living conditions, and peers. Socioenvironmental factors are distal factors and include culture and spoken dialect [1].

Several studies in Europe and the United States have discovered that HL is lower in older adults. HL is essential for older adults because it influences their physical and mental health, risk of cardiovascular disease, oral health, self-care, and even mortality [6,7]. HL research in Asia is mostly disease oriented and focused on the health knowledge of older patients with chronic diseases [8,9]. In addition, the influencing factors of HL are not well studied [10]. Improving HL and its assessment through designated HL environment and health policies could help the health sector improve the population’s expectations, preferences, and skills [11]. Therefore, identifying the influencing factors of HL among older adults is critical in Asia. Especially in older people, some socioenvironmental determinants (e.g., culture, language, etc.) or situational determinants (e.g., social support, family and peer influences, etc.) would deeply influence their access to or use of health-related information, and these determinants may be different from those in younger adults. Furthermore, especially in Chinese culture, older adults usually feel embarrassed when they are lacking certain knowledge or skills, which makes HL assessment in Far Eastern countries special and difficult [12].

In a Taiwanese study of self-perceived HL, advanced age and lower education level were associated with poorer perceived HL. Measuring self-perceived HL is simple, but self-perceived HL and actual functional HL differ [13]. HL involves not only the ability to access and understand medical information, it also involves analyzing and applying such information in diverse situations [5,6,7,8,9,10]. Identifying the strengths and weaknesses of people’s HL can help them develop personal health strategies or guide community health policies [14].

In Taiwan, health information leaflets and questionnaires are in Mandarin Chinese; however, older adults may be more accustomed to their familiar spoken dialect [15]. Therefore, based on the EU model and empirical evidence from existing literature, this study proposes the examination of personal (gender, age, education, past occupation, current job status, household income, and insurance status), situational (marital status, living situation, number of children, frequency of watching health-related television (TV) programs, frequency of attending health-related courses, and medical assistance), and socioenvironmental (dominant spoken dialect) factors influencing HL among older adults, with an emphasis on their spoken dialect.

## 2. Materials and Methods

### 2.1. Participants and Procedures

A cross-sectional survey was conducted at a district hospital and affiliated community center in northern Taiwan from August 2016 to May 2017. Participants were recruited through convenience sampling when they attended either physical therapy for musculoskeletal pain or community-based group exercise programs at the hospital. The inclusion criteria were (1) age of at least 65 years, (2) no cognitive impairment detected during Mini-Cog instrument screening [16], and (3) ability to communicate and complete a self-administered questionnaire.

This self-administered questionnaire consisted of items related to personal, situational, and socioenvironmental factors influencing HL, which included demographic information asked on the Taiwan National Health Interview Survey (NHIS) (e.g., birth year, gender, education levels, marriage status, residential area, past occupation, income, living status, dominant spoken dialect, etc.) [17]. If the participants could not complete the form because of illiteracy or poor eyesight, two trained interviewers conducted face-to-face interviews in the participants’ familiar dialect to collect the same information. Personal factors included gender, age, education, past occupation, current job status, household income, and private health insurance other than the National Health Insurance. Situational factors included marital status, living situation, number of children, frequency of watching health-related TV programs, frequency of attending health-related courses, and medical assistance (presence or absence of people providing support during doctor appointments or when making medical decisions). The socioenvironmental factor considered was the dominant spoken dialect (Hakka, Taiwanese, or Mandarin). This study was approved by the Institutional Review Board of Taoyuan Hospital (TYGH105004) on 7 November 2016.

### 2.2. Measures

This study used the Mandarin Chinese version of the European Health Literacy Survey Questionnaire (HLS-EU-Q47). The HLS-EU-Q47 has shown good psychometric properties [18,19]. The questionnaire consists of 47 items assessing three domains (health care, disease prevention, and health promotion) and four levels of HL assessed through questions about access to, understanding of, appraisal of, and application of health information. Responses to each item of the questionnaire are from 1 (very difficult) to 4 (very easy) [1]. To easily compare the three domains of health, the scores of all participants’ HL items were summed and standardized to a unified HL index from 0 to 50, where the index = (mean − 1) × (50/3), as recommended by the European Health Literacy Project. Higher HL index indicates more adequate HL [20]. The HLS-EU-Q47 defines four levels of HL with this index: 0 to 25 is inadequate HL, 25 to 33 is problematic, 33 to 42 is sufficient, and 42 to 50 is excellent. The Mandarin Chinese version of the HLS-EU-Q47 has been validated in an older adult population [13] and in patients with chronic diseases in Taiwan [21,22].

### 2.3. Statistics

One-way analysis of variance was performed to compare the HL associated with demographic variables. Hierarchical linear regression analysis was used to identify the influencing factors of HL for older adults. We designed three models based on the three domains of HL. Model 1 assesses personal factors, namely gender, age, education, past occupation, and insurance status. Model 2 incorporates situational factors, namely marital status, community involvement, medical assistance, health-related TV watching, and health-related course attendance. Model 3 adds spoken dialect (i.e., the socioenvironmental factor). The sample size of this study was based on a previous study that reported an adjusted *R*^2^ of 17.4% with type I error of <0.05 and power of 0.8. Thus, we required at fewest 100 older adults and 14 determinants: gender, age, education, past occupation, job status, household income, insurance status, marital status, living condition, number of children, health-related TV viewing, health-related course attendance, medical assistance, and dominant spoken dialect. All statistical analyses were performed using IBM SPSS version 22.0. Significance was defined as *p* < 0.05.

## 3. Results

### 3.1. Demographic Variables

A total of 161 older adults were recruited. Among them, 34 (21.1%) participants completed the questionnaire through verbally administered survey by the interviewers. The average age was 75.16 years (standard deviation = 6.53), 41.3% had less than six years of educational attainment, and 11.20% were uneducated. More than half had worked as physical laborers in the past (60.8%). Regarding household income, 51.2% of participants’ households earned 20,000 to 50,000 New Taiwan dollars (NTD) per month. For comparison, in 2017, the mean and median monthly incomes for the Taiwanese population were 49,989 and 39,166 NTD, respectively (i.e., 1615 and 1265 USD, respectively). Only 17.8% of the participants often watched health-related TV programs. The majority of older adults lived with others (83.8%) (Table 1). A plurality of participants spoke Hakka (44.7%) as their dominant dialect, followed by Mandarin (33.5%) and Taiwanese (21.7%).

### 3.2. Health Literacy

Overall, the mean HL index in this study was 30.83. The percentages of participants with inadequate, problematic, sufficient, and excellent HL were 22.98%, 34.78%, 29.19%, and 13.04%, respectively (Table 2). Limited HL, that is, inadequate or problematic, accounted for 57.76% of participants.

### 3.3. Influencing Factors of HL

Table 3 presents the personal determinants related to HL (*R*^2^ = 0.36) among older adults. In Model 1, significant influencing factors were age ≥85 years (*β* = −9.08, *p* < 0.001), 1–6 years of educational attainment (*β* = 5.25, *p* = 0.024), >6 years of educational attainment (*β* = 8.28, *p* < 0.001), and unknown insurance status (*β* = −8.06, *p* = 0.005). Model 2 additionally incorporated the situational factors explaining HL (*R*^2^ = 0.37). Significant factors were age >85 years (*β* = −7.86, *p* = 0.003), >6 years of educational attainment (*β* = 6.33, *p* = 0.002), and unknown insurance status (*β* = −9.89, *p =* 0.002). Model 3 added spoken dialect as a determinant of HL (*R*^2^ = 0.56), and significant factors were age >85 years (*β* = −8.50, *p* < 0.001), unknown insurance status (*β* = −9.04, *p* = 0.001), and dominant spoken dialect of Hakka (*β* = −10.28, *p* < 0.001) or Taiwanese (*β* = −8.34, *p* < 0.001).

## 4. Discussion

To our knowledge, this is the first study exploring the effect of different personal, situational, and socioenvironmental determinants of HL among older adults in Taiwan. Exploring the health-related skills of the aging population in Taiwan through HL levels may help guide policies to improve HL among older adults. For HL assessment, we used the HLS-EU-Q47, a comprehensive questionnaire assessing the broad concept of HL in terms of three domains (health care, disease prevention, and health promotion) and four competencies regarding health information (access, understanding, appraisal, and application).

### 4.1. HL of Older Adults

In the present study, more than half (57.6%) of the older adults had limited general HL, as well as limited HL in each of the questionnaire domains (health care, disease prevention, and health promotion). Sufficient and excellent HL were more prevalent in the health care and disease prevention domains than in the health promotion domain. An explanation is that data were collected in a hospital setting where older adults received more information regarding health care and disease prevention than regarding health promotion.

Older adults usually have low education levels, poor communication abilities, high morbidity rates, or functional limitations, which can influence their acquisition, understanding, or application of health information in making medical decisions or practicing self-care [23,24,25,26]. Therefore, improvement of the HL environment on the part of healthcare facilities by providing age-friendly navigation, written or oral communication, technology, and health policies for older adults could be a prerequisite solution for older adults with inadequate HL [27,28]. In addition, our results did not reveal a significant effect of past occupation, while a previous study mentioned that older adults who used to be laborers were shown to have lower HL [29].

### 4.2. Influencing Factors of HL

#### 4.2.1. Personal Factors

In our study, some personal factors (85+ years old, education, and insurance) were significantly associated with HL. However, neither past occupation nor current job status had a significant relationship with the level of HL. Some previous studies mentioned that patients’ HL may be related to their employment and job types [29,30], while another study proposed prior and current occupation had no association with older adults’ HL [31]. The possible reason may be that, as compared with other personal determinants, past or current occupation was a less relevant factor influencing older adults’ HL in the statistical model.

Older age groups were more likely to have poorer physical status and understanding [19], which may impair the ability to access, understand, appraise, and apply health information, and thus reduce HL. However, it was non-significant when situational factors or socioenvironmental factors were included. This may be because older adults can obtain health information by watching health-related TV programs [32] or from the Internet [33]. In our study, the observation of the poorest HL among adults aged ≥85 years might be related to education. When these adults were of school age, education was not widely mandatory or available in Taiwan; therefore, many older adults above 85 years old are illiterate and have inadequate HL. Moreover, approximately 5% of the study population who had lower HL were unsure whether they had purchased additional private insurance in addition to the Taiwan National Health Insurance, which is a mandatory single-payer system that covers more than 99% of Taiwan’s population since 1995. It also agrees with an Australian study that people who actively purchased private health insurance had superior active health management, social support, and understanding of health information [34].

#### 4.2.2. Situational Factors

Our study suggested no significant association between situational factors and HL among older adults. A study of living situations in Taiwan revealed that more than 70% of older adults choose to live with their families [35]. However, in our study, over 90% (146 out of 161) of older adults lived with family members or someone else. They are prone to lower HL scores than their counterparts. They are often unable to seek medical treatment on their own and must rely on family members they live with. Consequently, they usually have poor medical decision-making abilities [36]. In addition, we assessed both the current job status and past occupations of the participants because a previous study mentioned that past experiences (e.g., relationships with other individuals or communities) were reported to affect the lives of aging adults [37]. However, occupation was not a significant determinant of HL in our study.

#### 4.2.3. Socioenvironmental Factors

Dominant spoken dialect is a major determinant of HL among older adults in our study. Appropriate communication in an adult’s dominant spoken dialect can improve personal awareness of the medical system, self-care skills, and HL [34,35,36,37,38]. We found Taiwanese- and Hakka-speaking older adults had significantly lower HL than those who spoke Mandarin. The reason might be that the questionnaire was administered in Mandarin, the most commonly written and spoken official language in Taiwan. This problem also resulted in difficulty attaining HL especially for older adults who mainly speak Taiwanese and Hakka. A study on immigrants concluded that their HL was impeded by their language and culture [39]. Therefore, healthcare professional should consider the HL of older adults and use the appropriate spoken dialect to disseminate health-related information and reduce health inequality.

### 4.3. Limitations

Our study has several limitations. First, this was a cross-sectional study. Therefore, causal relationships could not be established. We could not eliminate potential selection bias because of the convenience sampling method and small sample obtained from a single hospital and a community center. Second, the subjective assessment of the HLS-EU-Q47 is sensitive to the emotional state of participants and recall bias. In addition, about one-fifth of our participants completed the questionnaire through face-to-face interviews instead of self-administration, which might influence truthfulness about some sensitive issues, such as education and occupation. Third, our eligibility criteria excluded older adults who could not follow instructions to complete the assessment. Also, our study sample was from a single area and the sample size was not large. Thus, the results should be cautiously generalized to other age groups or living areas.

## 5. Conclusions

More than half of the older adults in this study had limited HL. Among the three domains of HL, Taiwanese older adults had higher HL in health care and disease prevention than in health promotion. We concluded that factors significantly associated with HL included age over 85 years old, dominant spoken dialect (Taiwanese or Hakka), and not knowing insurance status. Our study results may help clinicians’ early identification of older adults at high risk for poor HL and health administrators to establish geriatric health education and policies through establishing an age-friendly HL environment. From our findings, we recommend that it be tailored to individuals’ age, education level, and health insurance services, as well as their dominant dialects.

## Figures and Tables

**Table 1:** Descriptive statistics of health literacy (HL) and demographics.

Demographic Characteristics	*N*	(%)	HLS-EU						*p* Value
			Mean	(Lower–Upper Limits of 95% CI)					
**Personal factors**									
Gender									0.218
Male	80	51.2	31.79	(29.98–33.61)					
Female	81	48.8	29.88	(27.40–32.35)					
Age (years)									<0.001
65–74	79	54.4	34.16	(32.45–35.87)					
75–84	65	38.4	29.33	(26.98–31.67)					
≥85	17	7.2	21.08	(14.63–27.52)					
Education									<0.001
Illiterate	26	11.2	21.37	(16.88–25.86)					
1–6 years	67	41.3	30.59	(26.90–32.72)					
>6 years	68	47.5	34.68	(32.82–36.54)					
**Past occupation**									0.001
Ordinary staff	57	39.2	34.13	(32.12–36.14)					
Physical labor	104	60.8	65.87	(26.99–31.05)					
Current job status									0.108
Retired	105	63.3	29.92	(28.06–31.78)					
Still working	56	36.7	32.53	(29.82–35.24)					
**Household income (NTD/month)**									0.117
<20,000	35	23.1	32.69	(29.91–35.46)					
20,000–50,000	81	51.2	31.38	(29.42–33.34)					
>50,000	45	25.7	28.39	(24.73–32.05)					
Insurance status									<0.001
Private insurance	116	74.0	31.66	(30.15–33.16)					
National Health Insurance only	31	21.2	33.94	(30.72–37.16)					
Unknown	14	4.8	17.08	(9.28–24.88)					
**Situational factors**									
Marital status									0.513
Married	134	83.8	31.06	(29.39–32.72)					
Divorced or other	27	16.2	29.70	(25.63–33.77)					
Living situation									0.34
With others	146	83.9	30.59	(28.95–32.24)					
Alone	15	16.2	33.14	(29.31–36.98)					
Children									0.15
None, or children <15 years	21	14.3	33.71	(29.19–38.23)					
Children ≥15 years	140	85.7	30.40	(28.76–32.03)					
**Health-related TV programs**									0.004
Often	23	17.8	36.59	(33.60–39.57)					
Rarely	128	82.2	30.27	(28.55–32.00)					
**Health-related courses**									0.275
Often	13	8.8	33.69	(26.92–40.45)					
Rarely	148	91.2	30.58	(29.00–32.15)					
Medical assistance									0.005
No	133	82.6	29.83	(28.11–31.54)					
Yes	28	17.4	35.57	(32.70–38.44)					
**Socioenvironmental factor**									
Dominant spoken dialect									<0.001
Hakka	72	44.7	26.12	(23.68–28.56)					
Taiwanese	35	21.7	29.13	(26.97–31.29)					
Mandarin	54	33.5	38.21	(36.62–39.79)					

Abbreviations: HLS-EU, European Health Literacy Survey; NTD: New Taiwan dollar; TV: television; CI: confidence interval.

**Table 2:** General and domain-specific HL index and level (*N* = 161).

HL	Mean (SD)	Inadequate *N* (%)	Problematic *N* (%)	Sufficient *N* (%)	Excellent *N* (%)
General health literacy	30.83 (9.82)	37 (23.0)	56 (34.8)	47 (29.2)	21 (13.0)
Health care	31.16 (8.52)	35 (21.7)	53 (32.9)	60 (37.3)	13 (8.1)
Disease prevention	31.43 (11.21)	30 (18.6)	54 (33.5)	46 (28.6)	31 (19.3)
Health promotion	29.94 (11.42)	45 (28.0)	43 (26.7)	52 (32.3)	21 (13.1)

Abbreviations: HL, health literacy; SD, standard deviation. Scores (under each level) of all participants’ HL items are in parentheses.

**Table 3:** Influencing factors of health literacy in hierarchical linear regression models.

Influencing Factors of HL	Model 1			Model 2			Model 3		
	β	95% CI	*p* Value	β	95% CI	*p* Value	β	95% CI	*p* Value
**Personal factors**									
Gender									
Male (ref.)									.
Female	0.08	(−2.79, 2.95)	0.956	0.43	(−2.58, 3.45)	0.776	0.37	(−2.16, 2.90)	0.771
Age (years)									
65–74 (ref.)									
75–84	−0.90	(−4.25, 2.45)	0.595	−0.28	(−4.05, 3.49)	0.884	−2.16	(−5.35, 1.04)	0.185
≥85	−9.08	(−13.73, −4.43)	0.000	−7.87	(−12.98, 2.74)	0.003	−8.50	(−12.80, 4.19)	<0.001
Education									
Illiterate (ref.)									
1–6 years	5.25	(0.69, 9.74)	0.024	3.36	(−1.51, 8.22)	0.175	2.06	(−2.04, 6.15)	0.322
>6 years	8.28	(3.32, 13.24)	0.001	6.33	(0.77, 11.88)	0.026	3.49	(−1.22, 8.21)	0.145
Past occupation									
Physical labor (ref.)									
Ordinary staff	−1.08	(−6.88, 4.71)	0.712	1.37	(−5.38, 8.12)	0.689	3.91	(−1.79, 9.61)	0.177
Current job status									
Retired (ref.)									
Still working	4.60	(−3.76, 12.95)	0.279	3.90	(−4.88, 12.68)	0.382	3.35	(−4.02, 10.73)	0.370
Household income (NTD/month)									
<20,000 (ref.)									
20,000–50,000	−0.50	(−3.87, 2.87)	0.770	0.22	(−3.33, 3.77)	0.902	−0.11	(−3.09, 2.86)	0.941
50,000–100,000	−2.02	(−6.35, 2.31)	0.358	−0.66	(−5.45, 4.13)	0.787	−0.46	(−4.47, 3.56)	0.822
>100,000	2.00	(−3.07, 7.06)	0.437	2.94	(−2.37, 8.24)	0.276	1.50	(−2.97, 5.98)	0.507
Insurance status									
National health insurance only (ref.)									
Private insurance	1.44	(−2.06, 4.94)	0.419	1.68	(−2.16, 5.52)	0.388	−0.17	(−3.42, 3.08)	0.918
Unknown	−8.06	(−13.67, −2.45)	0.005	−9.89	(−16.18, 3.61)	0.002	−9.04	(−14.35, 3.73)	0.001
**Situational factors**									
Marital status									
Married (ref.)									
Divorced or other				−0.65	(−5.12, 3.82)	0.774	1.88	(−1.96, 5.73)	0.335
Living situation									
With others (ref.)									
Alone				1.31	(−3.73, 6.35)	0.608	0.09	(−4.23, 4.42)	0.966
Children									
Children ≥15 years (ref.)									
None or children <15 years				2.60	(−1.55, 6.76)	0.217	1.63	(−1.87, 5.13)	0.359
Health-related TV programs									
Often (ref.)									
Rarely				−1.31	(−5.53, 2.91)	0.54	0.74	(−2.84, 4.31)	0.684
Health-related courses									
Often (ref.)									
Rarely				−1.26	(−6.77, 4.24)	0.651	−0.91	(−5.53, 3.71)	0.697
Medical assistance									
Yes (ref.)									
No				2.42	(−1.39, 6.24)	0.211	2.98	(−0.23, 6.19)	0.069
**Socioenvironmental factor**									
Dominant spoken dialect									
Mandarin (ref.)									
Hakka							−10.28	(−13.04, 7.51)	<0.001
Taiwanese							−8.34	(−11.52, 5.17)	<0.001
*R* ^2^		0.36			0.37			0.561	
Adjusted *R*^2^		0.31			0.28			0.153	

Abbreviations: ref., Reference. CI: confidence interval.

## References

[1] Sorensen K., Van den Broucke S., Fullam J., Doyle G., Pelikan J., Slonska Z., Brand H., Consortium Health Literacy Project European (2012). Health literacy and public health: A systematic review and integration of definitions and models. BMC Public Health.

[2] Nutbeam D. (2008). The evolving concept of health literacy. Soc. Sci. Med..

[3] MacLeod S., Musich S., Gulyas S., Cheng Y., Tkatch R., Cempellin D., Bhattarai G.R., Hawkins K., Yeh C.S. (2017). The impact of inadequate health literacy on patient satisfaction, healthcare utilization, and expenditures among older adults. Geriatr. Nurs..

[4] Sudore R.L., Yaffe K., Satterfield S., Harris T.B., Mehta K.M., Simonsick E.M., Newman A.B., Rosano C., Rooks R., Rubin S.M. (2006). Limited literacy and mortality in the elderly: The health, aging, and body composition study. J. Gen. Intern. Med..

[5] Jordan J.E., Buchbinder R., Osborne R.H. (2010). Conceptualising health literacy from the patient perspective. Patient Educ. Couns..

[6] Serper M., Patzer R.E., Curtis L.M., Smith S.G., O’Conor R., Baker D.W., Wolf M.S. (2014). Health literacy, cognitive ability, and functional health status among older adults. Health Serv. Res..

[7] Agarwal G., Habing K., Pirrie M., Angeles R., Marzanek F., Parascandalo J. (2018). Assessing health literacy among older adults living in subsidized housing: A cross-sectional study. Can. J. Public Health.

[8] Chen I.H., Chi M.J. (2015). Effects of self-care behaviors on medical utilization of the elderly with chronic diseases—A representative sample study. Arch. Gerontol. Geriatr..

[9] Tung H.-H., Lu T.-M., Chen L.-K., Liang S.-Y., Wu S.-F., Chu K.-H. (2014). Health literacy impact on elderly patients with heart failure in Taiwan. J. Clin. Gerontol. Geriatr..

[10] Sorensen K., Van den Broucke S., Pelikan J.M., Fullam J., Doyle G., Slonska Z., Kondilis B., Stoffels V., Osborne R.H., Brand H. (2013). Measuring health literacy in populations: Illuminating the design and development process of the European Health Literacy Survey Questionnaire (HLS-EU-Q). BMC Public Health.

[11] Rudd R., Anderson J. (2006). The Health Literacy Environment of Hospitals and Health Centers. Partners for Action: Making Your Healthcare Facility Literacy-Friendly.

[12] Dong P., Huang X.I., Wyer R.S. (2013). The illusion of saving face: How people symbolically cope with embarrassment. Psychol. Sci..

[13] Chung M.H., Chen L.K., Peng L.N., Chi M.J. (2015). Development and validation of the health literacy assessment tool for older people in Taiwan: Potential impacts of cultural differences. Arch. Gerontol. Geriatr..

[14] Batterham R.W., Hawkins M., Collins P.A., Buchbinder R., Osborne R.H. (2016). Health literacy: Applying current concepts to improve health services and reduce health inequalities. Public Health.

[15] Lee S.Y., Tsai T.I., Tsai Y.W., Kuo K.N. (2010). Health literacy, health status, and healthcare utilization of Taiwanese adults: Results from a national survey. BMC Public Health.

[16] Borson S., Scanlan J.M., Chen P., Ganguli M. (2003). The Mini-Cog as a screen for dementia: Validation in a population-based sample. J. Am. Geriatr. Soc..

[17] Shih Y.T., Hung Y.T., Chang H.Y., Liu J.P., Lin H.S., Chang M.C., Chang F.C., Hsiung C.A., Wu S.L. (2003). The design, contents, operation and the characteristics of the respondents of the 2001 National Health Interview Survey in Taiwan. Taiwan J. Public Health.

[18] Huang Y.J., Lin G.H., Lu W.S., Tam K.W., Chen C., Hou W.H., Hsieh C.L. (2018). Validation of the European Health Literacy Survey Questionnaire in Women with Breast Cancer. Cancer Nurs..

[19] Huang Y.J., Chen C.T., Lin G.H., Wu T.Y., Chen S.S., Lin L.F., Hou W.H., Hsieh C.L. (2018). Evaluating the European Health Literacy Survey Questionnaire in Patients with Stroke: A Latent Trait Analysis Using Rasch Modeling. Patient.

[20] Nakayama K., Osaka W., Togari T., Ishikawa H., Yonekura Y., Sekido A., Matsumoto M. (2015). Comprehensive health literacy in Japan is lower than in Europe: A validated Japanese-language assessment of health literacy. BMC Public Health.

[21] Hou W.H., Huang Y.J., Lee Y., Chen C.T., Lin G.H., Hsieh C.L. (2018). Validation of the Integrated Model of Health Literacy in Patients with Breast Cancer. Cancer Nurs..

[22] Huang S.C., Kuo S.F., Tsai P.S., Tsai C.Y., Chen S.S., Lin C.Y., Lin P.C., Hou W.H. (2020). Effectiveness of Tailored Rehabilitation Education in Improving the Health Literacy and Health Status of Postoperative Patients with Breast Cancer: A Randomized Controlled Trial. Cancer Nurs..

[23] Chen A.M., Yehle K.S., Albert N.M., Ferraro K.F., Mason H.L., Murawski M.M., Plake K.S. (2014). Relationships between health literacy and heart failure knowledge, self-efficacy, and self-care adherence. Res. Soc. Adm. Pharm..

[24] Heijmans M., Waverijn G., Rademakers J., van der Vaart R., Rijken M. (2015). Functional, communicative and critical health literacy of chronic disease patients and their importance for self-management. Patient Educ. Couns..

[25] Kobayashi L.C., Wardle J., Wolf M.S., von Wagner C. (2016). Aging and Functional Health Literacy: A Systematic Review and Meta-Analysis. J. Gerontol. B Psychol. Sci. Soc. Sci..

[26] Liu Y., Wang Y., Liang F., Chen Y., Liu L., Li Y., Yao H., Chu Q. (2015). The Health Literacy Status and Influencing Factors of Older Population in Xinjiang. Iran. J. Public Health.

[27] Harvard T.H. Chan School of Public Health. Health Literacy Studies Web Site. http://www.hsph.harvard.edu/healthliteracy.

[28] Groene R.O., Rudd R.E. (2011). Results of a feasibility study to assess the health literacy environment: Navigation, written, and oral communication in 10 hospitals in Catalonia, Spain. J. Commun. Healthc..

[29] Souza J.G., Apolinario D., Magaldi R.M., Busse A.L., Campora F., Jacob-Filho W. (2014). Functional health literacy and glycaemic control in older adults with type 2 diabetes: A cross-sectional study. BMJ Open.

[30] Mollakhalili H., Papi A., Zare-Farashbandi F., Sharifirad G., HasanZadeh A. (2014). A survey on health literacy of inpatient’s educational hospitals of Isfahan University of Medical Sciences in 2012. J. Educ. Health Promot..

[31] Ansari H., Almasi Z., Ansari-Moghaddam A., Mohammadi M., Peyvand M., Hajmohammadi M., Bagheri F. (2016). Health Literacy in Older Adults and Its Related Factors: A Cross-Sectional Study in Southeast Iran. Health Scope.

[32] Duong V.T., Lin I.F., Sorensen K., Pelikan J.M., Van Den Broucke S., Lin Y.C., Chang P.W. (2015). Health Literacy in Taiwan: A Population-Based Study. Asia Pac. J. Public Health.

[33] Sentell T. (2012). Implications for reform: Survey of California adults suggests low health literacy predicts likelihood of being uninsured. Health Aff..

[34] Beauchamp A., Buchbinder R., Dodson S., Batterham R.W., Elsworth G.R., McPhee C., Sparkes L., Hawkins M., Osborne R.H. (2015). Distribution of health literacy strengths and weaknesses across socio-demographic groups: A cross-sectional survey using the Health Literacy Questionnaire (HLQ). BMC Public Health.

[35] Liao M.Y., Yeh C.J., Lee S.H., Liao C.C., Lee M.C. (2018). Association of providing/receiving support on the mortality of older adults with different living arrangements in Taiwan: A longitudinal study on ageing. Ageing Soc..

[36] Shen H.N., Lin C.C., Hoffmann T., Tsai C.Y., Hou W.H., Kuo K.N. (2019). The relationship between health literacy and perceived shared decision making in patients with breast cancer. Patient Educ. Couns..

[37] Liu Y.B., Liu L., Li Y.F., Chen Y.L. (2015). Relationship between Health Literacy, Health-Related Behaviors and Health Status: A Survey of Elderly Chinese. Int. J. Environ. Res. Public Health.

[38] Altin S.V., Stock S. (2016). The impact of health literacy, patient-centered communication and shared decision-making on patients’ satisfaction with care received in German primary care practices. BMC Health Serv. Res..

[39] Tsai H.M., Cheng C.Y., Chang S.C., Yang Y.M., Wang H.H. (2014). Health literacy and health-promoting behaviors among multiethnic groups of women in Taiwan. J. Obstet. Gynecol. Neonatal. Nurs..

